# Seasonal variation in maternal dietary diversity is reduced by small‐scale irrigation practices: A longitudinal study

**DOI:** 10.1111/mcn.13297

**Published:** 2021-12-14

**Authors:** Kaleab Baye, Dawit Mekonnen, Jowel Choufani, Seid Yimam, Elizabeth Bryan, Jeffrey K. Grifith, Claudia Ringler

**Affiliations:** ^1^ Center for Food Science and Nutrition Addis Ababa University Addis Ababa Ethiopia; ^2^ Environment and Production Technology Division of the International Food Policy Research Institute Addis Ababa Ethiopia; ^3^ Environment and Production Technology Division of the International Food Policy Research Institute Washington District of Columbia USA; ^4^ Public Health and Community Medicine, Tufts University School of Medicine Tufts University Boston Massachusetts USA

**Keywords:** dietary diversity, irrigation, seasonality, sustainable intensification

## Abstract

Some agricultural practices, such as irrigation, have the potential to buffer seasonal dietary gaps and through increased production and consumption improve diets, particularly of the rural poor relying on subsistence farming but also for rural and urban households purchasing irrigated produce on local markets. The study aimed to evaluate the effect of seasonality and irrigation on women's diet in rural Ethiopia. Using a longitudinal study design, three rounds of surveys were conducted among women of reproductive age (15–49 years). Data on socioeconomic status, food consumption and haemoglobin concentration was collected. Energy and nutrient intakes were estimated using an interviewer‐administered multiple‐pass 24‐h recall. Women's dietary diversity score (WDDS), the proportion of women meeting the minimum dietary diversity for women (MDDW), haemoglobin concentration, the prevalence of anaemia and energy and nutrients intakes were compared between irrigators and nonirrigators and by season. Associations between MDDW/WDDS and irrigation status were assessed using fixed‐effect models, after adjusting for covariates. WDDS was low (3–4 out of 10 food groups) and exhibited high seasonal variability (*p* < 0.05). Diets were predominantly cereal‐based, with little consumption of nutrient‐dense foods like fruits and animal source foods. High seasonal variability in energy, protein, vitamin C, calcium, iron and zinc intakes were observed (*p* < 0.01). Irrigators were more likely to meet the MDDW than women from non‐irrigating households (*p* < 0.05). No cases of malaria were reported from the three rounds of screening. There is a high seasonal variation in women's diet, but this could be partly offset by irrigation practices.

## Key message

- Women's dietary diversity was low and exhibited high seasonal variation.
- The associations between season and diets were mediated by irrigation.
- Higher energy intakes were observed among women from households practising irrigation.

## Graphical abstract

High seasonal variability in energy, protein, vitamin C, calcium, iron and zinc intakes were observed. The high seasonal variation in women's diet could be partly offset by irrigation practices.

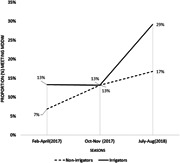

## 1 INTRODUCTION

Poor nutrition affects one‐third of the world's population and is now the highest risk factor associated with the global burden of disease (Forouzanfar et al., 2016; World Health Organization, 2017). The highest burden of undernutrition is found in low‐ and middle‐income countries (LMIC), with Africa south of the Sahara and South Asia being the most affected regions (UNICEF‐WHO‐WB, 2019). Poor diets, both in quantity and quality, along with diseases are key underlying proximal causes of malnutrition (Webb et al., 2018). Women of reproductive age and children in LMIC are disproportionately affected. Furthermore, the prevalence of undernutrition is often higher in rural than in urban communities.

According to the most recent Ethiopian demographic and health survey, 22.4% of women are undernourished as reflected by a low body mass index (BMI < 18.5; CSA, 2016). In rural Ethiopia, seasonal and chronic undernutrition is exacerbated by an over‐reliance on rain‐fed agriculture that is increasingly becoming unpredictable. In addition, poor market linkages and limited basic infrastructure (e.g., electricity) make nutrient‐dense perishable foods less accessible and affordable (Hirvonen et al., 2016). The Ethiopian government has recently increased efforts to expand irrigation, which, if implemented well, could increase dietary diversity, increase micronutrient intake and prevent anaemia by filling energy and nutrient intake deficits through increased availability and accessibility of nutrient‐dense foods (Keiser et al., 2005; Passarelli et al., 2018). However, irrigation water has also been linked to vector‐borne diseases such as malaria, which can adversely affect nutritional status (Kibret et al., 2014).

While the seasonality of households' and children's diets is well documented, little evidence exists regarding the seasonality of women's diets in Africa (Arsenault et al., 2014; Savy et al., 2006; Waswa et al., 2021). Moreover, whether irrigation practices bridge seasonal dietary gaps and eventually improve nutrition remains largely unknown. Recent studies suggest that irrigation can improve nutrition indirectly, through changes in production, income, water, sanitation and hygiene environment and women's empowerment (Domènech, 2015; Passarelli et al., 2018). However, the evidence remains scant and concerns over increased incidence of malaria have also been reported (Kibret et al., 2014). The lack of research on irrigation‐nutrition pathways is unfortunate given the Ethiopian governments' investments in irrigation. In 2020, the Ethiopian Irrigation Development Commission announced its plan to undertake nine irrigation projects covering 125,000 hectares and with the aim of ensuring household food security. This is on top of at least 13 ongoing large‐scale irrigation development projects with a combined command area of more than 400,000 hectares that were under construction as of early 2019 (Ministry of Agriculture of Ethiopia, personal communications, February 22, 2019). The 2018 National Smallholder Irrigation and Drainage Strategy of Ethiopia estimate around 7 million hectares of land to be economically viable to be irrigated by small holders in the country (GoE, 2018). However, with adequate planning the irrigation schemes could also be designed to improve nutrition. This study aims to build the evidence base to provide insight on nutrition‐sensitive irrigation development, using panel data on women's diets from three rounds of surveys at different periods during the production calendar. It aims to evaluate women's diet across seasons and how it may be influenced by irrigation practice.

## 2 DATA AND METHODS

### 2.1 Study sites, design and study participants

The study was conducted as part of the Sustainably Intensified Production Systems Impact on Nutrition' (SIPS‐IN) program funded by the USAID Feed the Future Sustainable Intensification Innovation Lab, which aims to evaluate the impact of irrigated production systems on food security, nutrition and health outcomes. The SIPS‐IN program took place in the watersheds of Robit and Dangila districts, Amhara region, in northern Ethiopia. These sites were selected given their high potential for irrigation based on an ex‐ante analysis of factors such as groundwater availability, distance to surface water and market access as well as interest to participate in irrigation activities by farming communities. Within these sites, households were randomly selected using the following criteria: produced crops were permanent residents of Robit or Dangila districts, and had at least one child less than 5 years of age. A longitudinal study consisting of household‐level and individual‐level surveys was conducted, and the same women 15–49 years of age (*n* = 364) were surveyed three times: in February–April 2017 (short rainy season—*belg*), October–November, 2017 (harvest) and July–August, 2018 (rainy season—*meher*). All the women self‐reported that they were nonpregnant at the time of the surveys. February–April (Season 1) represented an irrigation season, which also was a major fasting season (Lent fasting) for the Ethiopian Orthodox Church followers that accounted for 99% of our sample. The fasting season lasted 55 days preceding Easter. During this time, observers skip meals (breakfast and sometimes lunch) and adopt a vegan diet that is devoid of all animal source foods. The months of October and November (Season 2) are harvest seasons, where produces from the main rainy season are ready for household consumption, irrespective of access to irrigation. The months of July–August (Season 3) coincide with the lean season, when food availability is the lowest for nonirrigators. Considering the means and standard deviations of the women's dietary diversity score (WDDS) and energy intakes and *α* at 0.05, our post‐hoc analyses using GPower software suggested that our sample size had a power of 99% (two‐tailed test).

### 2.2 Data

#### 2.2.1 Sociodemographic and household characteristics

Information on the sociodemographic characteristics of the study participants was collected using pretested questionnaires that included questions about the age of household members, family size, education level, livelihood strategy and land size. The participant's flow chart is presented in Figure S1.

#### 2.2.2 Irrigation

Irrigation status was defined for each season depending on whether the household has at least one irrigated plot in the production season during the recall period. The study included areas where large irrigation systems are rarely available in the communities. Our survey data shows that irrigator households obtain water using hand buckets or hose, diesel pumps, hand or foot pumps and gravity methods. However, the dominant irrigation methods are hand buckets or hose (65.8%) and diesel pumps (33.8%).

#### 2.2.3 Dietary intake assessment

Using a quantitative 24‐h recall (1‐day recall), food intake was estimated using the multiple‐pass technique validated for use in developing countries (Gibson & Ferguson, 2008). The first‐pass was to identify all foods and drinks consumed in the last 24 h; the second pass was to identify ingredients and processing techniques applied in preparing the foods. The third pass is to make portion size estimations. To this end, whenever possible, salted‐replicas of actual foods were weighed using kitchen scales (Kinlee ACS‐EK01); or else, local utensils and graduated models were used. The final‐pass was to check the completeness of the data gathered. All days of the week were equally represented in the final sample.

The foods consumed were categorized into the following 10 nutritionally relevant food groups, according to the guide for measuring dietary diversity for women (Martin‐Prevel et al., 2017): (1) grains, white roots and tubers and plantains; (2) pulses (beans, peas and lentils); (3) nuts and seeds; (4) dairy; (5) meat, poultry and fish; (6) eggs; (7) dark green leafy vegetables (DG‐LV); (8) other vitamin A‐rich fruits and vegetables; (9) other vegetables; and (10) other fruits. The mean WDDS was calculated and the proportion of women that consumed at least 5 of the 10 food groups were considered to have met the minimum dietary diversity for women (MDDW) (Martin‐Prevel et al., 2017).

#### 2.2.4 Estimation of energy and nutrient intakes

Food intake was converted to energy and nutrient intakes using the Nutrisurvey software and linking food intake to food composition values from the Ethiopian food composition table that included nutrient composition data of local recipes (EHNRI, 1998). Missing values were completed using either published literature (Abebe et al., 2007; Umeta et al., 2005) or data from the USDA nutrient database (https://ndb.nal.usda.gov/ndb/), after adjusting for moisture difference. For mixed dishes, energy and nutrient contributions were calculated from local recipes. The detailed procedures followed are described elsewhere (Gibson & Ferguson, 2008). Intakes (per day) of energy (kcal), protein (g), vitamin A (µg RE), vitamin C (mg), calcium (mg), iron (mg) and zinc (mg) were estimated.

#### 2.2.5 Haemoglobin and malaria screening

Haemoglobin concentration was determined using a portable photometer (Hemocue HB 301; Ängelholm) from a blood sample from fingerpicks. The haemoglobin readings were adjusted for altitude (average 1900 m above sea level) and were categorized into nonanaemic and anaemic (any anaemia). Women with adjusted haemoglobin concentrations <12 g/dl were considered to be anaemic (WHO, 2011) and were referred to local health centres. Screening for malaria was performed using a rapid diagnostic test.

### 2.3 Ethics

Ethical approval was obtained by the Human Ethics Committees of Texas A&M (ref# 2014‐301D and amendment ref# IRB 2017‐0060D), the International Food Policy Research Institute (IRB #00007490 then renewed IRB #00007490)and the Amhara National Regional State Health Bureau Research Ethics Committee (HRTT/01/671/09, renewed HRTT03/973/2010 EC) in Ethiopia. All questionnaires and consent forms were translated to *Amharic* before the survey and written consent was obtained from study participants.

### 2.4 Quality control

Data were collected electronically using tablets and Survey CTO software. All survey rounds were conducted by the same experienced and trained enumerators. The enumerators were professionals with at least a Bachelors' degree, fluent in *Amharic* and whose main occupation was survey data collection in various regions of Ethiopia. One‐week training was provided before the first‐round survey, and refreshers were given just before the second and third round surveys were conducted.

### 2.5 Statistical analyses

All continuous variables were checked for normality using the Kolmogorov–Smirnov test. The dietary intake data were entered and processed using Nutrisurvey 2007 http://www.nutrisurvey.de/. Medians and interquartile range (first and third quartiles) of energy and nutrient intakes were calculated. The relationship between seasons and dietary diversity was assessed by fitting a season × irrigation interaction term in the regression model.

We used generalized estimating equations model for comparing dietary diversity, haemoglobin concentrations and energy and nutrient intakes between irrigators and nonirrigators and by season.

We also run fixed effect panel models with MDDW, WDDS, as well as women's vitamin C, calcium and vitamin A intakes as dependent variables and adjusting for time‐variant confounders and time‐invariant individual fixed effects. The general econometric model can be described as follows:

(1) $$y_{\text{it}}={ß}_{0}+{ß}_{k}X_{k,\text{it}}+a_{i}+e_{\text{it}},$$

where, *y*_it_ represents the dependent variables (MDDW or WDDS, Vit‐A, calcium and Vit‐C), where MDDW takes a value of 1 for women who met the minimum dietary diversity, WDDS takes a value between 0 and 10, and vitamin A, calcium and vitamin C are the natural logarithmic transformations of vitamin C, calcium and vitamin A intakes; *X*_k,it_ represent time varying socioeconomic characteristics, irrigation, season of the survey, irrigation‐season interactions, size of land ownership, number of adults in the household, number of children under 5 years of age, off‐farm income, orthodox fasting and lactation; *ß*_k_ is a matrix of coefficients for the respective explanatory variables; *a*_i_ represents all observed and unobserved individual time‐invariant characteristics that can be reasonably expected to be fixed over the three survey seasons; and *e*_it_ are individual time‐variant error terms. We run the models for MDDW and WDDS with and without the interaction between irrigation status and survey season. We run the fixed effects by clustering the standard errors at the village level to address the issue of possible clustering of results within a village. The decision to irrigate by individuals is not random by its nature and there is a selection bias. However, this decision is mainly affected by individual time‐invariant characteristics such as having land closer to a water source and individual‐specific factors such as work–ethic, risk‐taking behaviour, social and economic connections and so forth. These factors are not going to change meaningfully over the course of the 2 years between surveys and thus can be accounted for by a household fixed effect. We run a fixed effect regression that washes out/accounted for individual time‐invariant factors, which allowed us to remove the main sources of selection bias.

## 3 RESULTS

A total of 364 women were recruited for the study, and ∼95% completed all three rounds. However, partial noncompliance reached 18% in the third round, for relatively invasive procedures such as finger‐pricks for anaemia screening. The average land size owned by a household was 0.36 hectare, with a significant proportion (45%–67%) of households having access to irrigation at different periods of the year (Table 1). About 70% of households owned latrines, whereas 28.7% had no toilet facility. The average family size was 6. The mean age of the women was 39 years old, well over half had no formal education and only 30% had some primary‐level education. The household characteristics by irrigation status are presented in Table S1.

**Table 1:** Sociodemographic characteristics of study participants

Household/women's characteristics (*n* = 364)	*N* (%)
Land size owned (in hectare)	0.36 ± 0.1
Has access to irrigated plots	
Feb–Apr 2017	45.7%
Oct–Nov 2017	67.2%
Jul–Aug 2018	61.9%
No toilet facility	28.7%
Pit latrine with slab	68.5%
Ventilated improved pit latrine	<1%
Family size (mean ± SD)	5.8 ± 0.2
Mean age of women (years)	39.3 ± 11.2
Women with some level of primary education	30.0%
Women with some level of secondary education	4.2%

*Note*: Values are mean ± standard deviations or frequencies (%).

**Figure 1:**
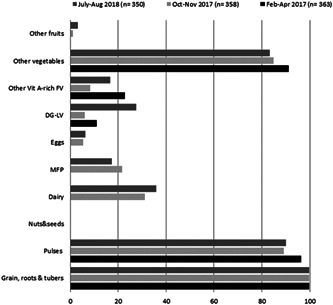
Food groups consumed by women in the 24 h preceding the survey, by season. FV, fruits and vegetables; MFP, meat, fish and poultry

The most widely consumed food groups by women were cereals, followed by pulses, and ‘other vegetables’ (Figure 1). The consumption levels of more nutrient‐dense food groups like eggs, dairy, DG‐LV, meat, fish and poultry (MFP) were low and showed high seasonal variability. For example, the consumption of animal‐source foods like eggs, MFP and dairy were extremely low (<1%) in Round 1 (February–April 2017), which corresponded with the Orthodox fasting season, but then gradually increased in the subsequent rounds. Consumption of fruits was very low, while nuts and seeds were absent from the diets of the women.

**Figure 2:**
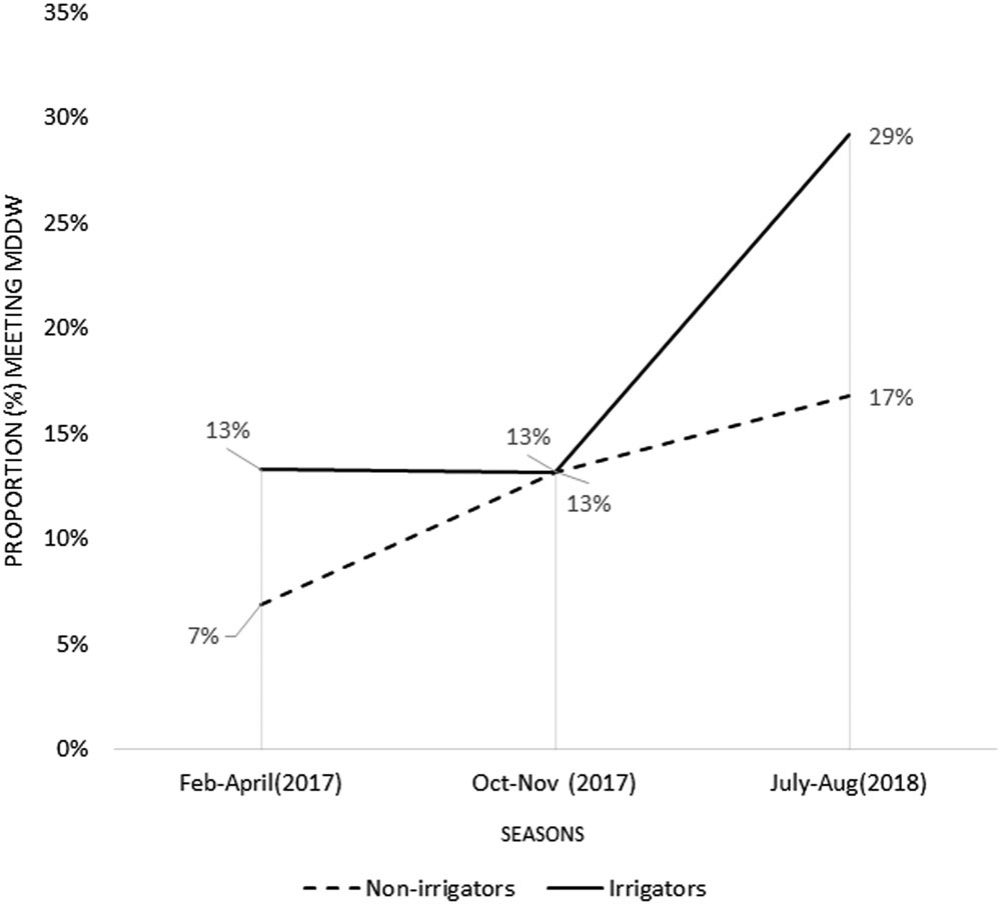
The proportion of women meeting the minimum dietary diversity score for women (MDDW) in the 24 h preceding the survey, by season and irrigation status

Overall, WDDS was very low (3–4 food groups out of 10), but with some variation by season and household irrigation access (*p* < 0.05; Table 2). The proportion of women meeting the MDDW, corresponding to the consumption of 5 out of 10 food groups, ranged from 10% in February–April 2017, to 23% in July–August, 2018 (*p* < 0.05). The proportion of women meeting the MDDW was slightly higher among irrigators than nonirrigators in February–April and July‐August rounds (Figure 2). The prevalence of anaemia showed statistically significant seasonal variation (Table S3).

**Table 2:** Women's dietary diversity, by irrigation and season

	All	Irrigators (no. of observations in parenthesis)	Nonirrigators (no. of observations in parenthesis)	*p*‐value^a^ (irrigation)	*p*‐value^b^ (season)
WDDS (mean ± SD)					
Feb–Apr 2017 (*n* = 363)	3.1 ± 0.6	3.3 ± 0.7 (166)	3.0 ± 0.6 (197)	0.013	
Oct–Nov 2017 (*n* = 360)	3.5 ± 0.9	3.6 ± 0.9 (242)	3.5 ± 0.8 (118)		<0.001
Jul–Aug 2018 (*n* = 342)	3.8 ± 1.1	3.9 ± 1.1 (212)	3.9 ± 0.9 (130)		
Proportion meeting MDDW (%)					
Feb–Apr 2017 (*n* = 363)	10.4%	12.7% (166)	8.6% (197)	0.089	
Oct–Nov 2017 (*n* = 360)	13.7%	14.1% (242)	12.7% (118)		<0.001
Jul–Aug 2018 (*n* = 342)	23.6%	27.4% (212)	19.3% (130)		

*Note*: Values are mean ± SD or %.

Abbreviations: MDDW, minimum dietary diversity for women; WDDS, women's dietary diversity score.

^a^ *p*‐value is from between‐subject comparison using general estimating equations with irrigation status as a dependent variable.

^b^ *p*‐value is from within‐subject comparison using general estimating equations with season (survey round) as a dependent variable.

Table 3 presents the energy and nutrient intake of women. High seasonal variability in the intakes of energy, protein, vitamin C, calcium, iron and zinc were observed (*p* < 0.01). Energy and calcium intakes were significantly higher among irrigators than nonirrigators (*p* < 0.05). However, the differences in vitamin C and calcium intakes between irrigators and nonirrigators were no more significant after adjusting for confounding factors (Table S2).

**Table 3:** Energy and nutrient intakes among irrigators and nonirrigators and by season (*N* = 327)

	Irrigators	Nonirrigators	*p*‐value^a^ (irrigation)	*p*‐value^b^ (season)
Energy (kcal)				
Feb–Apr 2017	1697.8 (1209.7, 2200.5)	1545.6 (1017.6, 2096.5)		
Oct–Nov 2017	2279.5 (1750.3, 2756.3)	2273.6 (1724.3, 2627.4)	0.041	<0.001
Jul–Aug 2018	1943.6 (1610.8, 2289.9)	1994.0 (1691.4, 2349.8)		
*All rounds*	1983.5 (1542.2, 2485.4)	1851.1 (1422.9, 2366.0)		
Protein (g)				
Feb–Apr 2017	47.5 (31.2, 58.6)	41.3 (26.7, 53.7)		
Oct–Nov 2017	58.2 (45.7, 73.9)	57.0 (46.3, 71.1)	0.357	<0.001
Jul–Aug 2018	53.8 (42.7, 67.2)	57.6 (46.0, 71.0)		
*All rounds*	54.0 (41.5, 67.6)	49.6 (38.0, 64.3)		
Vitamin A (µg RAE)				
Feb–Apr 2017	30.0 (17.5, 41.1)	27.9 (15.7, 42.1)		
Oct–Nov 2017	25.7 (5.4, 53.4)	28.1 (5.0, 57.3)	0.603	0.631
Jul–Aug 2018	15.0 (7.6, 28.1)	15.7 (8.8, 36.2)		
*All rounds*	21.1 (8.4, 42.6)	9.1 (4.7, 17.2)		
Vitamin C (mg)				
Feb–Apr 2017	15.1 (7.6, 22.8)	12.3 (6.7, 20.4)		
Oct–Nov 2017	33.0 (24.1, 43.8)	31.8 (22.7, 40.7)	0.082	<0.001
Jul–Aug 2018	32.0 (24.7, 42.1)	33.3 (24.5, 42.2)		
*All rounds*	28.7 (18.0, 39.5)	24.8 (12.1, 37.6)		
Calcium (mg)				
Feb–Apr 2017	926.7 (508.2, 1404.8)	682.6 (350.3, 1152.1)		
Oct–Nov 2017	789.1 (621.7, 1030.1)	765.5 (556.0, 932.5)	0.006	<0.001
Jul–Aug 2018	840.9 (634.6, 1120.7)	939.6 (745.2, 1157.5)		
*All rounds*	820.7 (621.3, 1133.5)	818.7 (528.7, 1085.5)		
Iron (mg)				
Feb–Apr 2017	128.6 (80.7, 182.6)	110.7 (60.5, 176.3)		
Oct–Nov 2017	162.5 (123.0, 228.5)	141.2 (99.0, 194.4)	0.209	0.003
Jul–Aug 2018	148.9 (99.0, 187.3)	160.3 (101.6, 198.1)		
*All rounds*	149.7 (103.5, 197.6)	134.4 (79.6, 190.5)		
Zinc (mg)				
Feb–Apr 2017	6.6 (4.7, 8.6)	5.8 (3.6, 7.8)		
Oct–Nov 2017	9.4 (7.2, 11.6)	9.4 (7.5, 11.7)	0.911	<0.001
Jul–Aug 2018	7.7 (6.4, 9.5)	8.1 (6.7, 10.4)		
*All rounds*	8.0 (6.3, 10.3)	7.5 (5.3, 9.8)		

*Note*: Energy and nutrient intakes are median (first quartile and third quartile) obtained from interview‐administered 24‐h recalls; intake data were complete for *n* = 327 for all the three rounds.

^a^ *p*‐values is from between‐subject comparison using general estimating equations.

^b^ *p*‐values are from within‐subject comparisons using GEE models with season (survey round) as a dependent variable.

Table 4 presents the regression model using MDDW and WDDS as outcome variables and controlling for various confounders. Both MDDW and WDDS are significantly higher among irrigators than nonirrigators in the February–April and July–August seasons compared with the reference season of October–November. February–April is an irrigation season as well as a major fasting season of 8 weeks in which followers of Ethiopian Orthodox Tewahedo Church, who make up almost all of our sampled women, abstain from animal source foods and many skip meals up to noon or 3 pm. The proportion of irrigating women who met the MDDW is twice that of women in nonirrigating households in this fasting season.

**Table 4:** Regression models predicting minimum dietary diversity for women (MDDW) and women's dietary diversity score (WDDS) and adjusting for confounders^a^

	(1)	(2)	(3)	(4)
	MDDW	WDDS	MDDW	WDDS
Irrigation status (1 = yes)^b^	0.040 (0.080)	0.344* (0.204)	−0.067 (0.085)	0.131 (0.225)
Feb–Apr 2017	0.134*** (0.033)	0.202** (0.090)	0.052 (0.045)	0.022 (0.121)
Jul–Aug 2018	0.079** (0.034)	0.311*** (0.082)	−0.004 (0.042)	0.149 (0.107)
Off‐farm income (1 = yes)	0.059 (0.040)	0.212** (0.097)	0.066* (0.039)	0.225** (0.096)
HH members age <= 5	0.021 (0.028)	0.094 (0.066)	0.023 (0.027)	0.111* (0.067)
Number of adults	0.005 (0.010)	0.040* (0.024)	0.004 (0.010)	0.038 (0.024)
Dummy for fasting (1 = yes)	−0.218*** (0.033)	−0.713*** (0.087)	−0.209*** (0.033)	−0.695*** (0.088)
Lactating (1 = yes)	0.061 (0.045)	0.114 (0.101)	0.065 (0.045)	0.123 (0.099)
Nonirrigated plot size (ha)	0.016 (0.039)	0.056 (0.101)	0.012 (0.039)	0.051 (0.101)
Irrigators *x* Feb–Apr 2017			0.121** (0.055)	0.269** (0.130)
Irrigators *x* Jul–Aug 2018			0.145** (0.061)	0.281* (0.150)
Constant	0.104 (0.082)	3.221*** (0.203)	0.170** (0.084)	3.351*** (0.214)
Observations	1000	1002	1000	1002

^a^ Regression models predicting MDDW and WDDS, adjusting for off‐farm income, number of children under 5 years of age, number of adults in household, lactation, size on nonirrigated land and unobserved time‐invariant household fixed effects; values are *β* coefficients (standard errors).

^b^ The reference is the Oct–Nov 2017 season with an ‘irrigation status, 1 = yes’; Models (3) and (4) include irrigation and season interactions.

* *p* < .10

** *p* < .05

*** *p* < .01.

In the October–November season, this corresponds to the harvest season from the main rainy season, the proportion of nonirrigating women who met MDDW pars with women from irrigating households (*p* > .05). In the lean season of July and August, the proportion of women who met the MDDW was again much higher in irrigating households than nonirrigating households. Fasting was strongly and negatively associated with both MDDW and WDDS in all the specifications (with and without the interaction of seasons and irrigation status). Survey rounds February–April, and July–August fare better in MDDW and WDDS than the October–November season, only in the models without the interactions of irrigation and seasons, as most of the seasonal variation of diets comes from households with irrigation. Such differences by season and irrigation status were not observed for anaemia and haemoglobin concentrations (Table S3).

## 4 DISCUSSION

The present study illustrates that women's dietary diversity was low and exhibited high seasonal variation. Diets were predominantly plant‐based with little consumption of nutrient‐dense food groups, such as animal source foods. High seasonal variability in energy, protein, vitamin C, calcium, iron and zinc intakes were observed. Women from irrigating households had higher WDDS and were more likely to meet the MDDW in February–April and July–August seasons, than women from nonirrigating households. No cases of malaria were reported in the three rounds. The associations between season and diets were mediated by irrigation.

Multiple reasons can explain the high seasonality of the diets of the women. Primarily, subsistence farming households rely on their own harvests that, in the absence of irrigation, happen once or twice a year (Sibhatu & Qaim, 2017). In addition to the limits of relying on rain‐fed production, poor market access, high price, limited food processing activities that extend the shelf‐life of perishable goods (Abay & Hirvonen, 2017; Gebru et al., 2018), high price fluctuations (Bachewe et al., 2017; Gilbert et al., 2017) and religious fasting further contribute to the seasonality of diets and consequently to varying levels of energy and nutrient intakes. Such seasonal dietary patterns have been commonly reported from other parts of rural Africa (Caswell et al., 2018; Savy et al., 2006), and can have serious adverse effects, including insufficient weight gain during pregnancy and low‐birthweight of babies (Rogawski McQuade et al., 2019; Toe et al., 2015).

The lowest energy intake, lowest animal‐source foods consumption and most meagre overall dietary diversity were found during the first round of the survey (February–April). This is unsurprising given that this period corresponded to the longest period of lent fasting (55 days), a period when observers reduce the number of meals and adopt a vegan diet. The majority of our study subjects are orthodox Christians. Consequently, a slightly higher consumption of vegetables accompanied by higher calcium and vitamin C intake was observed, but consumption of fruits remained low. Given that this period is in the dry season, unless households practice irrigation or have access to affordable nutrient‐dense foods like vegetables and fruits grown elsewhere, their diets are likely to remain poor in micronutrients. Noteworthy is also the food culture in rural Ethiopia, where leafy vegetables are often considered as a hunger food, and thus their consumption increases either during the fasting or the lean season, and drops once cereals and pulses become available. Consequently, the largest effect of irrigation in MDDW and WDDS is seen during the fasting and lean season and not during the harvest season. This seasonality in diets implies the need for bold efforts to improve data quality by capturing multiple time‐points within a year.

Women from households that practised irrigation had a higher dietary diversity than nonirrigating households. In the model adjusting for covariates, vitamin C and calcium intakes were also higher among women from households practising irrigation (Table S1). This is similar to findings on household dietary diversity in Ethiopia (Hirvonen et al., 2016). The statistically significant interaction between irrigation and season illustrates the buffering effect that irrigation can have on the seasonality of diets and energy/nutrient intakes. Multiple pathways including income, production and women's empowerment can explain these effects, but further studies are needed to decipher this.

Interestingly, no cases of malaria were reported in the three rounds of the survey, irrespective of smallholder irrigation practice. This supports recent reports of very low levels of malaria in the Amhara region (Yalew et al., 2017), but also indicates that smallholder irrigation—unlike larger‐scale irrigation systems with canal systems and higher likelihood of standing water—is not associated with high malaria incidence.

The strengths of the present study include the longitudinal study design that captured the dry and the rainy seasons, as well as the explicit evaluation of nutrition outcomes. The use of quantitative 24‐h recall data to estimate energy and nutrient intake across multiple seasons is also a key strength of the study. However, a second day repeat was not available to adjust for the within‐subject variance. In addition, our study defined the use of irrigation on plots broadly; hence, whether outcomes vary by level and type of irrigation needs further investigation.

The present study illustrates the seasonal variation of women's diet in rural Ethiopia and shows that this seasonal variability can be partly offset by irrigation practice. Higher energy intakes were observed among women from households practising irrigation. Irrigation schemes that fill the gap in nutrient‐dense foods (e.g., leafy vegetables) are likely to have more nutritional impact than those that aim to produce more cereals. Consequently, the Government of Ethiopia's plan to expand irrigation projects in the country could improve nutrition if oriented towards the production of nutrient‐dense foods. Future studies should investigate whether complementing irrigation interventions with nutrition‐related behavioural change communication would lead to a greater impact on improving diets and nutritional outcomes of women.

## CONFLICT OF INTERESTS

The authors declare that there are no conflict of interests.

## ETHICS STATEMENT

Ethical approval was obtained by the Human Ethics Committees of Texas A&M (ref# 2014‐301D and amendment ref# IRB 2017‐0060D), the International Food Policy Research Institute (IRB #00007490 then renewed IRB #00007490) and the Amhara National Regional State Health Bureau Research Ethics Committee (HRTT/01/671/09, renewed HRTT03/973/2010 EC) in Ethiopia.

## AUTHOR CONTRIBUTIONS

KB, DM, JC, EB, JKG and CR designed the research. KB, JC and EB conducted research. KB, DM, JC and SY analysed the data. KB, DM, EB and CR wrote the paper; all authors have read and approved the final draft of the manuscript.

## Supporting information

Supplementary information.

## Data Availability

The data that support the findings of this study are available from IFPRI, Environment and Production Technology Division, upon reasonable request
