# Supplementary material for: Seasonal variation in maternal dietary diversity is reduced by small‐scale irrigation practices: A longitudinal study
Source: Matern Child Nutr. 2021 Dec 14;18(2):e13297. doi: 10.1111/mcn.13297 (PMC8932846; doi:10.1111/mcn.13297)
Supplement: Supplementary file 1 — Supplementary information. [file MCN-18-e13297-s001.docx]

Non-irrigating (n=198)

Irrigating (n=166)

Excluded: Households without children under five years of age (N= 104)

Households in watersheds of Dangla and Robit districts enrolled in the Innovation Lab for Small Scale Irrigation (ILSI) project (N=368) and additionally recruited households under the SIPS project (N=100). In total, 468 households were recruited.

Eligible households recruited (N= 364)

Data available for analyses (N=166)

**Dietary diversity**

Round 1= 166

Round 2= 163

Round 3= 160 **Dietary intake** Round 1= 156

Round 2= 161

Round 3= 159

**Hemoglobin concentration**

Round 1= 157

Round 2= 155

Round 3= 137

Data available for analyses (N=198)

**Dietary diversity**

Round 1= 197

Round 2= 195

Round 3= 192 **Dietary intake** Round 1= 194

Round 2= 195

Round 3= 189

**Hemoglobin concentration**

Round 1= 190

Round 2= 190

Round 3= 167

Lost to follow-up Deceased (n=1)

Figure S1: Participant flow chart

Table S1 Summary statistics of main household characteristics by irrigation status

| Indicators | Non-irrigators (N=445) | Irrigators (N=620) | dif | p_value |
| --- | --- | --- | --- | --- |
| Household size | 5.391 | 5.723 | 0.332 | 0.008 |
| Number of adults (full working capacity) | 3.05 | 3.102 | 0.052 | 0.539 |
| Education of the main female member (years) | 0.963 | 1.252 | 0.289 | 0.056 |
| Land size (in hectare) | 1.807 | 1.887 | 0.08 | 0.189 |
| Total off-farm income of the household (ETB) | 1813.671 | 10276.44 | 8462.769 | 0.345 |
| Lactating (1=yes) | 0.27 | 0.36 | 0.09 | 0.002 |
| Altitude | 1946.008 | 1918.276 | -27.732 | 0.035 |

**TABLE S2** Regression model predicting Vitamin C, Calcium and Vitamin A intakes^1^

|  | **(1)** | **(2)** | **(3)** |
| --- | --- | --- | --- |
| **Outcomes^2^** | **ln_Vit C** | **ln_calcium** | **ln_Vit A** |
| Irrigation status (1=yes)^3^ | -0.031 | 0.102 | -0.054 |
|  | (0.143) | (0.137) | (0.310) |
| Feb-Apr 2017 | -1.103^***^ (0.103) | -0.189^*^ (0.097) | 0.536^**^ (0.211) |
| Jul-Aug 2018 | 0.0198 | 0.179^***^ | -0.207 |
|  | (0.070) | (0.065) | (0.190) |
| Irrigators *x* Feb-Apr 2017 | 0.225^*^ | 0.147 | -0.0734 |
|  | (0.123) | (0.114) | (0.222) |
| Irrigators *x* Jul-Aug 2018 | -0.078 | -0.099 | -0.095 |
|  | (0.097) | (0.078) | (0.236) |
| Constant | 3.462*** (0.140) | 6.491*** (0.134) | 3.199*** (0.294) |
| Observations (n) | 978 | 979 | 966 |

^1^Regression models predicting MDDW and WDDS, adjusting for off-farm income, number of children under 5 years of age, number of adults in household, lactation, size on non-irrigated land, and unobserved time-invariant household fixed effects; values are Beta coefficients (standard errors);

^2^ ln_Vit C, ln_calcium, and ln_Vit A refer to the natural logarithmic transformations of Vitamin C, Calcium, and Vitamin A intakes;

^3^The reference is the Oct-Nov 2017 season with an “irrigation status, 1=yes”; All models in this table include irrigation and season interactions;

^*^ *p* < 0.10, ^**^ *p* < 0.05, ^***^ *p* < 0.01;

**Table S3:** Regression models predicting Anemia occurrence and hemoglobin level^†^

|  | (1) | (2) | (3) | (4) |
| --- | --- | --- | --- | --- |
|  | Adjusted anemia (dummy) | ln(adjusted Hb) | Adjusted anemia (dummy) | ln(adjusted Hb) |
| Irrigation status (1=irrigators)^£^ | -0.0126 | -0.00811 | -0.00832 | -0.0112 |
|  | (0.0868) | (0.0175) | (0.1009) | (0.0200) |
|  |  |  |  |  |
| Feb-Apr 2017 | -0.00604 | -0.000192 | -0.0154 | 0.0000641 |
|  | (0.0428) | (0.0082) | (0.0612) | (0.0120) |
|  |  |  |  |  |
| July-Aug 2018 | -0.0535^*^ | 0.0109^*^ | -0.0445 | 0.00722 |
|  | (0.0312) | (0.0060) | (0.0519) | (0.0103) |
|  |  |  |  |  |
| Off farm income (1==Yes,0=No) | 0.0343 | 0.00820 | 0.0336 | 0.00845 |
|  | (0.0438) | (0.0088) | (0.0436) | (0.0087) |
|  |  |  |  |  |
| Number of adults (full working capacity) | -0.0394^*^ | 0.00520 | -0.0392^*^ | 0.00514 |
|  | (0.0224) | (0.0047) | (0.0225) | (0.0047) |
|  |  |  |  |  |
| Dummy for fasting (1=fasting) | -0.0289 | 0.0118 | -0.0303 | 0.0123 |
|  | (0.0458) | (0.0082) | (0.0461) | (0.0083) |
|  |  |  |  |  |
| Lactating (1=yes) | -0.0380 | 0.0142^*^ | -0.0375 | 0.0143^*^ |
|  | (0.0418) | (0.0081) | (0.0418) | (0.0081) |
|  |  |  |  |  |
| Non-irrigated plot size (hectare) | 0.0253 | -0.00753 | 0.0272 | -0.00802 |
|  | (0.0415) | (0.0084) | (0.0422) | (0.0086) |
|  |  |  |  |  |
| Irrigators *x* Feb-Apr 2017 |  |  | 0.0186 | -0.00151 |
|  |  |  | (0.0630) | (0.0116) |
|  |  |  |  |  |
| Irrigators *x* Jul-Aug 2018 |  |  | -0.0197 | 0.00716 |
|  |  |  | (0.0669) | (0.0137) |
|  |  |  |  |  |
| Constant | 0.440^***^ | 2.506^***^ | 0.435^***^ | 2.508^***^ |
|  | (0.1092) | (0.0227) | (0.1161) | (0.0241) |
| Observations | 955 | 955 | 955 | 955 |

^†^Regression models predicting the occurrence of adjusted anemia and adjusted hemoglobin level, controlling off-farm income, number of children under 5 years of age, number of adults in household, lactation, size on non-irrigated land, and unobserved time-invariant household fixed effects; values are Beta coefficients (standard errors in bracket);

^£^The reference is the Oct-Nov 2017 season with an “irrigation status, 1=yes”; Models (3) and (4) include irrigation and season interactions; ^*^ *p* < 0.10, ^**^ *p* < 0.05, ^***^ *p* < 0.01
